# The delayed response network: towards a single layer universal neural network approximator and delay-based learning

**DOI:** 10.1186/1471-2202-16-S1-P214

**Published:** 2015-12-18

**Authors:** Dinov Martin, Elias Rut

**Affiliations:** 1 Computational, Cognitive and Clinical Neuroimaging Laboratory, Imperial College London, London, UK; 2 Vienna University of Technology, Vienna, Austria

## 

**Figure 1:**
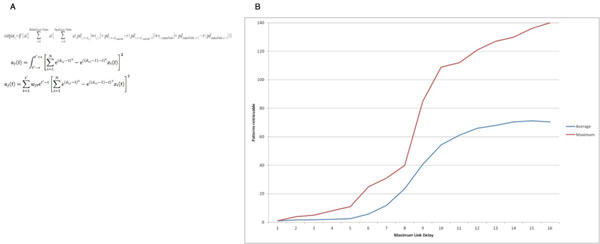
**A shows three different mathematical formulations implemented**. Top equation is original one with which exact bit patterns were learned, as implemented in Scala code. Bottom two equations of A were variations used in more recent work, solving XOR and classifying data. B shows a plot of amount of bit patterns learned by a 2-3-1 DRN architecture as implemented by the top equation in A, showing the effect of the maximum allowed delay per link/connection between two nodes (on x-axis) and the number of bits learned (average and maximum) on the y-axis.

We were interested in exploring the use of timing or delays in learning in neural networks. The work was developed by wondering about the effects of glia on network learning in the human brain. To investigate this idea, we created a Multilayer Perceptron (MLP)-based [1] feedforward model that included a signal propagation time (delay) parameter, which allowed for bit patterns to be learned exactly using only signal delay changes while keeping constant weights in simulations implemented in Scala (top equation in Figure 1a). We then changed the model to produce network architectures with a single combined hidden-output layer, by making the activation function a double-Gaussian and by summing (or integrating) the squared output from this layer in time (bottom two equations in Figure 1a). This allows for mutual inhibition between inputs and solution of the XOR problem. In each model, the delays are not used to give history to the function and expand the input space, as is common in time series prediction using ANNs. Instead, by either explicitly (implementation of top equation of Figure 1a) or implicitly (bottom two equations in Figure 1a) summing through time, we allow for the possibility of using delays beyond this input space expansion use. We show appropriately trained DRNs (bottom equations of Figure 1a), where all training is on the delay parameters, can successfully classify on some standard classification datasets. In such a feedforward context, we see that delay modifications can have a functional equivalence to weight modifications. We propose such feedforward delay-using ANNs as biologically more interesting ANNs that allow for further hypothesis formation and testing of learning and computational mechanisms in biological neural nets. We show that the delay space size (equivalently, time to compute a given function) has an effect on the number of unique bit patterns that can be learned (Figure 1b) and on the complexity of functions that can be modeled (e.g. for classification). Though the models are still caricatures of biological networks, they support and suggest a few important ideas: a) that signal propagation time changes can serve as a mechanism for learning in biological networks. As per our initial thoughts, these models support the notion that glial cells might, in vivo, implement such a delay-based learning mechanism. We believe glia have all the required mechanisms and couplings for doing this, for example via activity-dependent myelination modifications [2,3]. Also b) that the maximum waiting-time for a given region for a signal to arrive from another determines how sensitive the regions are to inter-region delay changes (e.g. on the axonal tracts), and, equivalently, how complex the functional coupling between the two regions can be and d) that the models show clear tradeoffs between complexity (or size) of a network, and the time required in computing a given function. This space-time tradeoff is akin to the many other tradeoffs and competing optimizations co-existing in the brain [4].
